# Acid and Fungus

**Published:** 1884-03

**Authors:** 


### Acid and Fungus.

Of the experiments of Dr. W. D. Miller, of Berlin, *The Independent Practitioner* for December has this from a letter but recently received from him:

"I have discovered and successfully cultivated a fungus *always* present in the human mouth and in carious dentine, whose development is, under proper conditions (such as are constantly found in the mouth), always accompanied by a strong acid. I have also determined the conditions favorable and unfavorable to its development, and have nearly finished the analysis of its acid. To ascertain this I have made over three hundred experiments and cultures. I am very hopeful that this will furnish a solution to the whole problem."

